# American tertiary clinic-referred bipolar II disorder versus bipolar I disorder associated with hastened depressive recurrence

**DOI:** 10.1186/s40345-017-0072-x

**Published:** 2017-01-25

**Authors:** Bernardo Dell’Osso, Saloni Shah, Dennis Do, Laura D. Yuen, Farnaz Hooshmand, Po W. Wang, Shefali Miller, Terence A. Ketter

**Affiliations:** 1 Department of Psychiatry, University of Milan, Fondazione IRCCS Ca’Granda, Ospedale Maggiore Policlinico, Milan, Italy; 2 Department of Psychiatry and Behavioral Sciences, Stanford University School of Medicine, Stanford, CA USA

**Keywords:** Bipolar disorder, Bipolar subtype, Time to depressive recurrence, Time to recovery

## Abstract

**Background:**

Bipolar disorder (BD) is a chronic, frequently comorbid condition characterized by high rates of mood episode recurrence and suicidality. Little is known about prospective longitudinal characterization of BD type II (BD II) versus type I (BD I) in relation to time to depressive recurrence and recovery from major depressive episode. We therefore assessed times to depressive recurrence/recovery in tertiary clinic-referred BD II versus I patients.

**Methods:**

Outpatients referred to Stanford BD Clinic during 2000–2011 were assessed with Systematic Treatment Enhancement Program for BD (STEP-BD) Affective Disorders Evaluation and with Clinical Monitoring Form during up to 2 years of naturalistic treatment. Prevalence and clinical correlates of bipolar subtype in recovered (euthymic ≥8 weeks) and depressed patients were assessed. Kaplan–Meier analyses assessed the relationships between bipolar subtype and longitudinal depressive severity, and Cox proportional hazard analyses assessed the potential mediators.

**Results:**

BD II versus BD I was less common among 105 recovered (39.0 vs. 61.0%, *p* = 0.03) and more common among 153 depressed (61.4 vs. 38.6%, *p* = 0.006) patients. Among recovered patients, BD II was associated with 6/25 (24.0%) baseline unfavorable illness characteristics/mood symptoms/psychotropics and hastened depressive recurrence (*p* = 0.015). Among depressed patients, BD II was associated with 8/25 (33.0%) baseline unfavorable illness characteristics/mood symptoms/psychotropics, but only non-significantly associated with delayed depressive recovery.

**Conclusions:**

BD II versus BD I was significantly associated with current depression and hastened depressive recurrence, but only non-significantly associated with delayed depressive recovery. Research on bipolar subtype relationships with depressive recurrence/recovery is warranted to enhance clinical management of BD patients.

## Background

Bipolar disorder (BD) comprises a spectrum of different mood disorders, characterized by variable severity of illness, functional impairment, comorbidity patterns, suicidal risk, cognitive impairment, and treatment response (Akiskal et al. 2000; Alda 2004; Duffy 2014). Within the bipolar spectrum, BD type II (BD II) and I (BD I) are currently the main clinically defined expressions of BD, as acknowledged by the fifth edition of the diagnostic and statistical manual of mental disorders (DSM-5) (American Psychiatric Association 2013).

In view of the phenomenological and biological heterogeneity of BDs and related clinical expressions, the recurrent episodic course has been traditionally considered the hallmark of the illness and the largest source of longitudinal burden (Angst and Sellaro 2000; Angst and Gamma 2008). Quality of remissions and frequency of recurrences are known to vary substantially between patients. Nevertheless, mood episode recurrences were found to occur in approximately half of bipolar patients within 2 years (Vazquez et al. 2015), and in approximately three-quarters within 5 years (Gitlin et al. 1995), despite continuous guideline-based treatment.

Among mood episode recurrences, there is a well-established predominance of depressive, compared with hypomanic/manic/mixed episodes, over the course of BD, with a subsequent overall greater burden in terms of economic costs, functioning, caregiver burden, and suicide (Judd et al. 2005; Di Marzo et al. 2006; Miller et al. 2014). For instance, a Systematic Treatment Enhancement Program for BD (STEP-BD) report showed that, among the 58% of patients who recovered from a syndromal mood episode at enrollment, approximately half had mood episode recurrence within 2 years, with twice as many depressive compared to mood elevation recurrences (Perlis et al. 2006).

More recently, in a systematic comparison of naturalistic studies versus randomized controlled trials, recurrence rates in BD were found to be substantial and similar, with first-episode recurrence-polarity being more often depressive than mood elevation (Vázquez et al. 2015). In addition, depressive compared to manic recurrences were found to not only be more frequent and prolonged (Perlis et al. 2006; Tondo et al. 2016), but also associated with greater multi-dimensional impairment and symptom severity (Di Marzo et al. 2006). Therefore, the frequency and severity of depressive recurrences in BD highlight the importance to identify clinical variables associated with hastened depressive recurrence (i.e., shorter time to next major depressive episode) in order to devise more effective disease management strategies.

Clinical variables associated with recurrences in BD include younger onset age and rapid cycling (Vázquez et al. 2015), poor sleep quality (Cretu et al. 2016), lifetime comorbid anxiety disorders and current anxiety (Otto et al. 2006; Shah et al. 2017), seasonality (Geoffroy et al. 2014), duration of recovery (Coryell et al. 1995), residual symptoms (Judd et al. 2008), subsequent treatment resistance, psychosocial disability, and possible functional neuroanatomic changes (Goldberg et al. 2005). In this perspective, the role of diagnostic subtype in relation to recurrences and achievement of recovery in bipolar patients has not shown significant associations with specific variables to date, particularly with respect to time to depressive recurrences and recovery in BD II versus BD I patients. In addition, while more studies have investigated recurrence rates in BD, as synthesized in a recent systematic review (Vázquez et al. 2015), less is known about time to depressive recovery and related mediators (Otto et al. 2006). In both cases, moreover, potential differences between BD II versus BD I patients have not been assessed, such investigation being of relevant clinical interest in order to implement specific therapeutic strategies in these subjects.

Aiming at assessing potential differences between BD subtypes, in a previous cross-sectional study by our group, we found in an American tertiary clinic-referred setting that BD II, compared to BD I patients, had illness that was more severe in multiple ways (e.g., more lifetime episodes, anxiety comorbidity, childhood onset, rapid cycling) but less severe in a few other ways (e.g., less hospitalizations and psychosis) (Dell’Osso et al. 2015). Following the above-mentioned investigation, the present study aimed to longitudinally assess BD subtype effects on time to depressive recurrence/recovery in patients with BD II versus BD I.

## Methods

We included outpatients with BD II or BD I referred by community practitioners (primarily psychiatrists) to the Stanford University BD Clinic between 2000 and 2011. Patients were assessed with the Systematic Treatment Enhancement Program for BD (STEP-BD) Affective Disorders Evaluation (Sachs et al. 2003), which included the Structured Clinical Interview for the Diagnostic and Statistical Manual of Mental Disorders, 4th edition (First et al. 1996) mood disorders module and Clinical Global Impression-Bipolar Version-Overall Severity (CGI-BP-OS) score (Spearing et al. 1997). The Mini International Neuropsychiatric Interview (MINI) (Sheehan et al. 1998) was used to confirm bipolar and comorbid psychiatric disorder diagnoses. Clinical status at each follow-up visit was determined by symptom ratings on the Clinical Monitoring form (CMF) (Sachs et al. 2002), while patients received measurement- and guideline-based naturalistic treatment (with monthly modal visit frequency) for up to 2 years.

Bipolar disorder subtype (BD II vs. BD I) was determined from available medical records and patient report, as assessed by the STEP-BD Affective Disorders Evaluation and MINI. Current mood symptoms were determined from patient report, as assessed by the STEP-BD Affective Disorders Evaluation at the time of enrollment, and clinician observation and reflected any mood symptoms in the 10 days prior to enrollment for the primary analysis, and mood symptoms thresholded for occurring on at least four or seven of the 10 days prior to enrollment for the secondary analysis. Current psychotropic medication use was based upon patient report, as assessed by the STEP-BD Affective Disorders Evaluation, and review of medical records at the time of enrollment. Antidepressants included Selective Serotonin Reuptake Inhibitors (SSRIs), Serotonin Norepinephrine Reuptake Inhibitors (SNRIs), Atypical Antidepressants (e.g., bupropion, mirtazapine), and First-Generation Antidepressants (e.g., heterocyclic antidepressants, monoamine oxidase inhibitors). Mood Stabilizers included lithium, valproate, carbamazepine, and lamotrigine. Antipsychotics included the second-generation agents olanzapine, risperidone, quetiapine, aripiprazole, and ziprasidone. Anxiolytic/hypnotics included benzodiazepine and non-benzodiazepine agents administered for anxiety (e.g., lorazepam, clonazepam, alprazolam, and buspirone) and/or insomnia (e.g., temazepam, zolpidem, and trazodone).

As described below, clinical characteristics of participants were evaluated, and prospective clinical course of participants meeting diagnostic criteria for either current recovery (euthymic ≥8 weeks) or depression (a current major depressive episode) at enrollment was assessed. The STEP-BD protocol and the subsequent similar Stanford-specific Assessment, Monitoring, and Centralized Database protocol were approved by the Stanford University Administrative Panel on Human Subjects, and patients provided verbal and written informed consent prior to participation. Trained medical and research staff collected data on six demographic parameters and 25 illness characteristics/current mood symptoms/current psychotropic medications. The demographic parameters assessed were (A) Age (in years); (B) Gender; (C) Race/Ethnicity; (D) Education; (E) Marital Status; and (F) Employment status. The illness characteristics/current mood symptoms/current psychotropic medications assessed were (1) lifetime anxiety disorder; (2) lifetime alcohol/substance use disorder; (3) lifetime eating disorder; (4) lifetime personality disorder; (5) bipolar II disorder; (5A) lifetime psychosis (which is very commonly associated with bipolar I disorder); (5B) lifetime prior psychiatric hospitalization (which is also very commonly associated with bipolar I disorder); (6) ≥one first-degree relative with mood disorder; (7) onset age (in years); (8) Childhood (age <13 years) onset; (9) illness duration (in years); (10) long illness duration (≥15 years); (11) episode accumulation (≥10 prior mood episodes); (12) lifetime suicide attempt; (13) rapid cycling (≥4 episodes) in prior year; (14) CGI-BP-OS; current (i.e., any in the prior 10 days) (15) sadness; (16) anhedonia; (17) euphoria; (18) irritability; and (19) anxiety; and current (baseline) (20) mood stabilizer use; (21) antipsychotic use; (22) antidepressant use; (23) anxiolytic/hypnotic use; (24) complex pharmacotherapy (≥4 mood stabilizers, antipsychotics, or antidepressants); and (25) number of core psychotropics (mood stabilizers, antipsychotics, or antidepressants).

Statistical analyses were performed using Statistical Package for the Social Sciences (SPSS) Version 23.0 software (IBM Corp.; Armonk, NY, USA) on an Apple MacBook Air computer (Apple Corporation, Cupertino, CA, USA). Prevalence and clinical correlates of BD II versus BD I were examined in currently recovered (i.e., euthymic ≥8 weeks) and currently depressed (i.e., with a current major depressive episode) patients. Analytical statistics included Fisher’s Exact test comparisons of categorical data and independent-sample *t* test comparisons of continuous variables. In addition, binary logistic regression was used to adjust for potential confounding variables. Primary longitudinal analyses consisted of Kaplan–Meier survival analyses (log-rank tests), which compared times to depressive recurrence and recovery in patients with BD II versus BD I. We used the standard approaches of censoring patients with mood elevation prior to depressive recurrence in assessing time to depressive recurrence, and censoring patients with depressive prior to mood elevation recurrence in assessing time to mood elevation recurrence (Tohen et al. 1990). Secondary metrics included for BD II versus BD I depressive longitudinal severity were Kaplan–Meier estimated recurrence/recovery rates for significant longitudinal depressive associations. Additional secondary analyses included Cox proportional hazard analyses [hazard ratios (HRs) and 95% confidence intervals (CIs)] for depressive recurrence and recovery, as well as for potential mediators of statistically significant longitudinal depressive illness severity findings. To select parameters for entry into mediator models, univariate Cox proportional hazard analyses were performed for all statistically significant clinical correlates of bipolar subtype. Parameters with *p* < 0.05 were entered into a forward stepwise procedure, and covariates were included in the model if *p* < 0.05. Additionally, Cox proportional hazard analyses with time-dependent covariates were used to further characterize statistically significant associations between bipolar subtype and depressive recurrence and recovery. To facilitate comparisons with prior studies, we also calculated observed and Kaplan–Meier estimated overall (all patients, any episode) recurrence/recovery rates. We used a two-tailed significance threshold with *p* < 0.05, with no correction for multiple comparisons.

## Results

Table 1 includes demographics, illness characteristics, and current mood symptoms/psychotropic medications of currently recovered and currently depressed patients, stratified by bipolar subtype. Our sample included 153 (30.4%) depressed and 105 (20.9%) recovered patients, drawn from 503 outpatients, with the remaining 245 patients being excluded for not satisfying inclusion criteria, due to lack of sustained recovery (i.e., euthymic <8 weeks; *N* = 102, 20.3%), presence of subsyndromal depression/mood elevation (i.e., >2 threshold level depressive or mood elevation symptoms, but NOT meeting DSM-IV criteria for a syndromal mood episode; *N* = 89, 17.7%), or syndromal mood elevation (i.e., hypomanic, manic, or mixed episodes; *N* = 54, 10.7%) clinical status.

**Table 1:** Demographics, illness characteristics, current mood symptoms, and current medications in bipolar II disorder versus bipolar I disorder outpatients

	Recovered bipolar II	Recovered bipolar I	Depressed bipolar II	Depressed bipolar I
*N* (%)	39.0* (41)	61.0 (64)	61.4** (94)	38.6 (59)
*Demographics*				
A. Age (years, mean ± SD)	36.5 ± 13.8	35.8 ± 13.7	36.5 ± 13.5	35.9 ± 13.5
B. Female (%)	69.0	48.4	63.8	57.6
C. Caucasian (%)	75.0	77.4	87.2	86.4
D. College degree (%)	66.7	60.3	51.1	40.7
E. Married (current, %)	34.1	38.1	41.5	30.5
F. Full-time employment (current, %)	35.0	33.3	25.8	25.4
*Comorbid disorders (lifetime, %)*				
1. Anxiety	61.0*	37.5	81.9	69.5
2. Alcohol/substance use	51.2	50.0	58.5	57.6
3. Eating	14.6	6.3	18.1	6.8
4. Personality	7.3	9.4	19.1*	6.8
*Other illness characteristics*				
5. Bipolar II disorder (%)	100.0****	0.0	100.0****	0.0
*5A. Psychosis (lifetime, %)*	*12.2*	68.8****	*18.1*	64.4****
*5B. Psychiatric Hospitalization (lifetime, %)*	*9.8*	70.3****	*6.4*	57.6****
6. ≥One 1° relative with mood disorder (%)	53.7	42.2	61.7	55.9
7. Onset age (years, mean ± SD)	18.4 ± 9.0	20.1 ± 8.8	16.2 ± 8.7	18.5 ± 5.8
8. Childhood (age <13 years) Onset (%)	20.0	9.4	31.9***	8.5
9. Illness duration (years, mean ± SD)	18.9 ± 15.4	16.4 ± 12.6	19.6 ± 13.5	17.3 ± 12.5
10. Long illness duration (≥15 years, %)	50.0	46.6	54.8	50.8
11. Episode accumulation (≥10, lifetime, %)	63.9	42.9	81.6**	56.4
12. Suicide attempt (lifetime, %)	30.8	23.8	31.2	39.0
13. Rapid cycling (prior year, %)	15.4	6.7	34.4	20.7
14. CGI-BP-OS (current, mean ± SD)	2.4 ± 0.7**	2.0 ± 0.8	5.4 ± 0.8	5.93 ± 0.7
*Current mood symptoms (any in prior 10* *days, %)*				
15. Sadness	31.7*	10.9	91.5	86.4
16. Anhedonia	22.4*	7.8	95.7	93.2
17. Euphoria	17.1	10.9	35.1*	16.9
18. Irritability	53.7**	25.0	77.7**	54.2
19. Anxiety	46.3	29.7	85.1	74.6
*Medication (current)*				
20. Mood stabilizer (MS, %)	75.6	77.8	51.1**	75.9
21. Antipsychotic (AP, %)	31.7	40.6	20.2****	55.9
22. Antidepressant (AD, %)	46.3*	25.0	48.9	37.3
23. Anxiolytic/hypnotic (AN, %)	24.4	23.4	31.9	40.7
24. Complex pharmacotherapy (>4 MS, AP, or AD, %)	24.4	15.6	24.5	35.6
25. Number of psychotropics (MS, AP, AD, mean ± SD)	2.6 ± 1.6	2.3 ± 1.3	2.2 ± 1.8*	2.8 ± 1.7

Underline indicates parameters with statistically significant relationships with bipolar subtype; CGI-BP-OS indicates clinical global impression for bipolar disorder-overall severity; italic font indicates parameters associated with bipolar I disorder, independent of mood state; SD indicates standard deviation

Missing data: recovered—≥10 prior episodes 12.4%, all other parameters 0.0–5.7%, depressed—≥10 prior episodes 7.2%, all other parameters 0.0–1.3%

* *p* < 0.05; ** *p* < 0.01; *** *p* < 0.001; **** *p* < 0.0001, bipolar II disorder versus bipolar I disorder

In our sample, BD II was significantly less common in recovered versus depressed patients (39.0 vs. 61.4%, Chi square = 12.5, *df* = 1, *p* = 0.0006).

### Demographics and illness characteristics/current mood symptoms/current psychotropic medications in recovered patients with bipolar II disorder versus bipolar I disorder

Among recovered patients, BD II compared to BD I was less common (39.0 vs. 61.0%, Binomial test, *p* = 0.031). Recovered patients with BD II versus BD I disorder had significantly higher rates of lifetime anxiety disorder (61.0 vs. 37.5%, Chi square = 5.5, *df* = 1, *p* = 0.027), BD II (by definition, 100.0 vs. 0.0%, Chi square = 105.0, *df* = 1, *p* < 0.0001), current sadness (31.7 vs. 10.9%, Chi square = 7.0, *df* = 1, *p* = 0.011), anhedonia (24.4 vs. 7.8%, Chi square = 5.6, *df* = 1, *p* = 0.023), irritability (53.7 vs. 25.0%, Chi square = 8.9, *df* = 1, *p* = 0.004), and antidepressant use (46.3 vs. 25.0%, Chi square = 5.1, *df* = 1, *p* = 0.034), as well as higher CGI-BP-OS (2.4 ± 0.7 vs. 2.0 ± 0.8, *t* = 2.9, *df* = 103, *p* = 0.004), but less prior psychosis (12.2 vs. 68.8%, Chi square = 32.1, *df* = 1, *p* < 0.0001) and prior psychiatric hospitalization (9.8 vs. 70.3%, Chi square = 36.8, *df* = 1, *p* < 0.0001), with the latter two being well-established BD II versus BD I differences, independent of mood state. Indeed, in our dataset these relationships were mediated by associations of BD I with prior psychosis and prior psychiatric hospitalization. In contrast, no assessed demographic parameter and no other illness characteristic/current mood symptom/current psychotropic medication used in Table 1 were significantly associated with BD II versus BD I among recovered patients.

### Demographics and illness characteristics/current mood symptoms/current psychotropic medications in depressed patients with bipolar II disorder versus bipolar I disorder

Among currently depressed patients, BD II compared to BD I was more common (61.4 vs. 38.6%, Binomial test, *p* = 0.006) and was associated with more lifetime personality disorder (19.1 vs. 6.8%, Chi square = 4.5, *df* = 1, *p* = 0.036), BD II (by definition, 100.0 vs. 0.0%, Chi square = 153.0, *df* = 1, *p* < 0.0001), childhood BD onset (31.9 vs. 8.5%, Chi square = 11.3, *df* = 1, *p* = 0.0007), ≥10 mood episode accumulation (81.6 vs. 56.4%, Chi square = 10.6, *df* = 1, *p* = 0.002), and current euphoria (35.1 vs. 16.9%, Chi square = 5.9, *df* = 1, *p* = 0.017) and irritability (77.7 vs. 54.2%, Chi square = 9.2, *df* = 1, *p* = 0.0039), but less current mood stabilizer (51.1 vs. 75.9%, Chi square = 9.2, *df* = 1, *p* = 0.0035) and antipsychotic (20.2 vs. 55.9%, Chi square = 20.6, *df* = 1, *p* < 0.0001) use, and fewer current psychotropic medications (2.2 ± 1.8 vs. 2.8 ± 1.7, *t* = 2.2, *df* = 151, *p* = 0.029), as well as less prior psychosis (18.1 vs. 64.4%, Chi square = 33.8, *df* = 1, *p* < 0.0001) and prior psychiatric hospitalization (6.4 vs. 57.6%, Chi square = 49.3, *df* = 1, *p* < 0.0001); However, the latter two relationships were mediated by associations of BD I with prior psychosis and prior psychiatric hospitalization (Table 1). In contrast, no assessed demographic parameter and no other illness characteristic/current mood symptom/current psychotropic medication used in Table 1 were significantly associated with BD II versus BD I among depressed patients.

### Bipolar subtype in relationship to time to and frequency of depressive recurrence

**Fig. 1:**
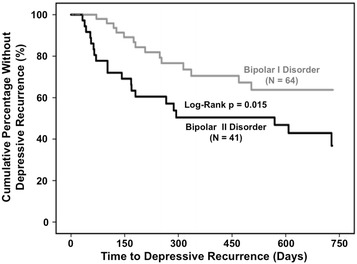
Bipolar II disorder associated with hastened depressive recurrence. Two-year survival analysis of time to depressive recurrence in recovered bipolar disorder patients indicated significantly hastened depressive recurrence in patients with bipolar II disorder (*N* = 41, *black line on bottom*) versus bipolar I disorder (*N* = 64, *gray line on top*, log-rank, *p* = 0.015). Bipolar II disorder compared to bipolar I disorder was also significantly associated with hastened depressive recurrence using Cox proportional hazard analysis [hazard ratio (HR) = 2.3 (95% confidence interval (CI) 1.2–4.5), *p* = 0.018]. Lifetime anxiety disorder (HR = 4.4, 95% CI 1.9–10.5, *p* = 0.001) drove and lifetime history of psychosis (HR = 0.24, 95% CI 0.088–0.68, *p* = 0.007) attenuated hastened depressive recurrence in patients with bipolar II disorder versus bipolar I disorder

BD II versus BD I was significantly associated with hastened depressive recurrence (log-rank, *p* = 0.015) in 41 versus 64 recovered patients (Fig. 1). BD II versus BD I was also significantly associated with hastened depressive recurrence using Cox proportional hazard analysis (HR = 2.3, 95% CI 1.2–4.6, *p* = 0.018). Hastened depressive recurrence among BD II versus BD I patients was driven by lifetime anxiety disorder (HR = 4.4, 95% CI 1.9–10.5, *p* = 0.001) and attenuated by lifetime history of psychosis (HR = 0.24, 95% CI 0.088–0.68, *p* = 0.007). BD II’s association with hastened depressive recurrence was significant in the first year (HR = 2.3, 95% CI 1.1–4.8, *p* = 0.027), again mediated by lifetime anxiety disorder (HR = 4.4, 95% CI 1.9–10.5, *p* = 0.001), and attenuated by lifetime psychosis history (HR = 0.24, 95% CI 0.088–0.67, *p* = 0.007), but not the second year (HR = 2.2, 95% CI 0.37–13.3, *p* = 0.39). BD II versus BD I patients had an only non-significantly higher (less than twice as high) 2-year Kaplan–Meier estimated depressive recurrence rate of 63.2% (95% CI 44.6–81.8) versus 36.2% (95% CI 20.5–51.9).

In contrast, associations between BD II versus BD I and times to mood elevation recurrence (log-rank, *p* = 0.13, HR = 0.44, 95% CI 0.14–1.3, *p* = 0.14, in 41 versus 64 recovered patients, not illustrated) and any mood episode recurrence (log-rank, *p* = 0.30, HR = 1.3, 95% CI 0.78–2.3, *p* = 0.30, in 41 versus 64 recovered patients, not illustrated) were non-significant. The observed and Kaplan–Meier estimated overall (all patients, any episode) 2-year recurrence rates were 50.5% and 65.9% (95% CI 54.9–76.9), respectively. As expected, the observed and Kaplan–Meier estimated overall (all patients, any episode) 1-year recurrence rates were lower at 41.0% and 50.6% (95% CI 39.8–61.4), respectively.

### Bipolar subtype in relationship to time to and frequency of depressive recovery

BD II versus BD I was not significantly associated with time to depressive recovery (log-rank, *p* = 0.35) in 94 versus 59 depressed patients (not illustrated). BD II versus BD I was also not significantly associated with time to depressive recovery using Cox proportional hazard analysis (HR = 1.2, 95% CI 0.79–1.95, *p* = 0.35) and was not significantly associated with rate of depressive recovery [86.2%, 95% CI (76.2–96.2) vs. 78.4%, 95% CI (63.6–93.2)]. In addition, BD II versus BD I was not significantly related to time to recovery from mood elevation episodes (log-rank, *p* = 0.28) or from any mood episode (log-rank, *p* = 0.35, not illustrated). The observed and Kaplan–Meier estimated overall (all patients, any episode) 2-year recovery rates were 46.4% and 84.3% (95% CI 76.5–92.1%), respectively. As expected, the observed and Kaplan–Meier estimated overall (all patients, any episode) 1-year recovery rates were lower at 37.2% and 59.5% (95% CI 50.5–68.5%), respectively.

## Discussion

To date and to our knowledge, the present study represents one of the first attempts to longitudinally assess differences in BD subtypes in relation to depressive recurrence/recovery in BD I versus BD II patients. Results showed that BD II versus BD I recovered patients showed significantly hastened depressive recurrence, driven by lifetime anxiety disorder and attenuated by prior psychosis. On the other hand, BD II versus BD I depressed patients had only a non-significant association with time to depressive recovery. Taken as a whole, our longitudinal and baseline comparison data, particularly in relation to the finding of hastened depressive recurrence in BD II patients, support, integrate, and extend previous reports by our and other groups, showing that BD II, at least in some clinical settings, is not merely a milder form of illness, compared to BD I (Vieta et al. 1997; Judd et al. 2003a, 2005; Novick et al. 2010; Dell’Osso et al. 2015, 2017).

Of note, findings on demographic and clinical variables showed that BD II versus BD I was less common among recovered subjects and more frequent among depressed patients, consistent with overall higher severity of illness, particularly in relation to depression proneness, for individuals with BD II versus BD I, as previously reported (Judd et al. 2003a, b; Dell’Osso et al. 2015; Faurholt-Jepsen et al. 2015).

Thus, our findings of recovered patients with BD II versus BD I having significantly greater burden of illness, with approximately one-quarter of baseline demographic and clinical variables, are consistent with available literature showing BD II-related increases of unfavorable baseline characteristics/current mood symptoms/current psychotropics, including higher rates of anxiety disorders (Rihmer et al. 2001; Henry et al. 2003; Dell’Osso et al. 2017), residual symptoms (Judd et al. 2003b) (specifically sadness, anhedonia and irritability in our sample), and antidepressant use (Born et al. 2009; Dell’Osso et al. 2017) in BD II versus BD I patients.

Furthermore, our findings on demographic and clinical variables in depressed patients indicating that BD II versus BD I patients had significantly greater problems with approximately one-third of baseline unfavorable illness characteristics/mood symptoms/psychotropics, including higher rates of lifetime personality disorders, childhood onset, >10 episode accumulation, current euphoria, and irritability, but less current mood stabilizer and antipsychotic use, and fewer current psychotropic medications, but less prior psychosis and psychiatric hospitalizations, are consistent with prior studies (Vieta et al. 1997; Dell’Osso et al. 2015; Yao et al. 2015).

The most noteworthy finding of the present study, however, is the longitudinal observation of hastened depressive recurrence in BD II versus BD I recovered individuals. This result, in fact, provides new insight regarding the well-established longitudinal vulnerability of BD II patients to depressive burden (Judd et al. 2003a, b; Dell’Osso et al. 2015). On one hand, our data indicate lifetime anxiety disorder as the mediator of hastened depressive recurrence in BD II versus BD I patients, supporting the well-established, mutual, and reciprocal relationship between BD and anxiety disorder comorbidity (Vázquez et al. 2014; Pavlova et al. 2015), with an additional longitudinal characterization. Of note, we found that hastened depressive recurrence in BD II versus BD I patients, mediated by lifetime anxiety disorder, was significant only in the first year of follow-up, which likely represents a crucial time-frame for the occurrence of depressive recurrences in BD II individuals, particularly in those with anxious comorbidity. Indeed, a previous Canadian study found recurrences were common after the first manic episode with more than one-half of the bipolar patients experiencing a mood event within 12 months, although differences between patients with BD II and BD I were not reported (Yatham et al. 2009).

On the other hand, our findings of lower rates of baseline prior psychosis and prior psychiatric hospitalization in both recovered and depressed BD II patients and of hastened depressive recurrence in BD II recovered individuals being attenuated by lifetime psychosis history further support the well-documented inverse and direct relationships between BD II and BD I, respectively, and prior psychosis and psychiatric hospitalization (Tohen et al. 1990).

Finally, our data on bipolar subtype in relation to time to and frequency of depressive recovery did not show any significant association, demonstrating the need for further investigation regarding this issue.

Longitudinal analysis on time to elevation mood episode and time to recovery from mood elevation episode did not show any significant difference in relation to BD subtype, highlighting the importance to futher characterize and differentiate BD II from BD I not only in light of the manic/hypomanic symptom dimension but, particularly, in terms of long-term depressive burden.

This study had noteworthy strengths and limitations. Strengths included assessing relationships between bipolar subtype and times to not only depressive recurrence but also depressive recovery, using validated instruments to assess diagnosis and longitudinal course, and having substantial numbers of both recovered (*N* = 105) and depressed (*N* = 153) well-characterized BD patients. In addition, our overall (all patients, any episode) recurrence/recovery rates were in broad agreement with prior studies (Vázquez et al. 2015; Otto et al. 2006; Perlis et al. 2006). Moreover, our findings were not merely the result of depression following episodes of mood elevation, since cases of patients with prior recurrences of manic, hypomanic, and mixed episodes were censored.

However, these strengths were accompanied by limitations that included the use of a sample referred to a suburban Northern California BD specialty clinic, limiting the generalizability of our findings in our relatively affluent, well educated but relatively underemployed, predominantly female sample of BD patients with medical insurance. Additionally, our sample size, though substantial, had insufficient statistical power to be able to assess the overall time spent in syndromal/symptomatic/non-symptomatic state in BD II versus I patients. Furthermore, recovered status/mood episode duration prior to enrollment was not included in our analyses of mood episode recurrence/recovery. Another limitation was the open naturalistic treatment design, in which patients received diverse uncontrolled (albeit guideline-informed and measurement- and evidence-based) interventions. In particular, baseline antidepressant use was significantly more common in patients with BD II compared to BD I. However, baseline antidepressant use was not a mediator of hastened depressive recurrence in patients with BD II versus BD I in our modeling, providing a more detailed assessment of the nature of the contribution of baseline pharmacotherapy, in general, and baseline antidepressant use, in particular, was beyond the scope of this study. Finally, we did not correct for multiple comparisons, which particularly limited interpretation of findings with *p* values between 0.05 and 0.01. However, this liberal statistical approach increased assay sensitivity with respect to our ability to detect relationships between bipolar subtype and baseline clinical characteristics as well as depressive recurrence/recovery.

Nevertheless, we contend that the association between BD II and hastened depressive recurrence suggests that BD II subtype entails an important vulnerability towards longitudinal depressive burden in BD. Given the large human and financial costs of depression in BD, further examination of relationships between bipolar subtype and depressive recurrence and recovery is warranted in order to enhance clinical management.

## Abbreviations

BD: bipolar disorder
BD I: bipolar disorder type I
BD II: bipolar disorder type II
CGI-BP-OS: clinical global impression for bipolar disorder-overall severity
CI: confidence interval
CMF: clinical monitoring form
df: degrees of freedom
HR: hazard ratio
MINI: mini international neuropsychiatric interview
SCID for DSM-IV: structured clinical interview for the diagnostic and statistical manual of mental disorders, IVth edition
SD: standard deviation
SPSS: Statistical Package for the Social Sciences
STEP-BD: systematic treatment enhancement program for bipolar disorder
